# Supplementary material for: The calcium binding protein S100β marks hedgehog-responsive resident vascular stem cells within vascular lesions
Source: NPJ Regen Med. 2021 Mar 1;6:10. doi: 10.1038/s41536-021-00120-8 (PMC7921434; doi:10.1038/s41536-021-00120-8)
Supplement: Supplementary file 1 — Supplentary Figures [file 41536_2021_120_MOESM1_ESM.pdf]

Supplementary Table I

Antibodies and their corresponding dilutions used in immunohistochemistry.

Antibody/Product Name	Supplier/Product Number	Dilutions
Rat anti-mouse Sca1/Ly6A/E antibody [D7] (FITC) antibody	Abcam (ab25031)	1/200
Rabbit anti-mouse/rat alpha smooth muscle cell α-actin antibody	Abcam (ab5694)	1/200
Anti-Patched / PTCH1 antibody, Mouse monoclonal	Abcam (Ab55629)	1/100
Anti-Actin, α-Smooth Muscle antibody, Mouse monoclonal	Sigma (A5228)	1/200
Anti-S100-β (CT) Antibody, clone EP1576Y, rabbit monoclonal	Millipore (04-1054)	1/100
Anti-Gli2 antibody, rabbit polyclonal	Novus Biologicals (NBP2-23602SS)	1/50
Chicken anti-GFP antibody	Abcam (ab13970)	1/1000
Rabbit Anti-RFP/dT antibody	Abcam (ab62341)	1/1000

Supplementary Table II

Antibodies and their corresponding dilutions used in immunocytochemistry/western blot.

Antibody/Product Name	Supplier/Product Number	Dilutions
Mouse anti-mouse/rat nestin [Rat-401]	Abcam (ab11306)	1/200
Rabbit anti-mouse Calponin [EP798Y]	Abcam (ab46794)	1/200
Goat anti-mouse/rat/human smooth muscle Myosin heavy chain	Santa Cruz (sc-79079)	1/200
Mouse anti-human/rat SOX10	R&D System (MAB2864)	1/100

Mouse anti-human SOX17	R&D System (MAB1924)	1/100
Rabbit Anti-S100β	Merck Millipore (ABN59)	1/100
Rabbit Anti-mouse/rat S100β [EP1576Y]	Abcam (ab52642)	1/100
Rabbit anti-mouse/rat Sca1	Millipore (AB4336)	1/100
Alexa Fluor® 488 Goat anti-mouse IgG	Invitrogen (A-11001)	1/1000
Alexa Fluor® 488 Goat anti-rabbit IgG	Invitrogen (A-11008)	1/1000
Alexa Fluor® 488 Donkey anti-goat IgG	Invitrogen (A-11055)	1/1000

Supplementary Table III

Antibodies used in Chromatin Immunoprecipitation (ChIP)

Antibody/Product Name	Supplier/Product Number
Rabbit anti-mouse Tri-Methyl-Histone H3 (Lys27) [C36B11]	Cell Signalling Technology (9733S)
Rabbit anti-mouse Di-Methyl-Histone H3 (Lys4) [C64G9]	Cell Signalling Technology (9725S)
Normal Rabbit IgG (ChIP graded)	Cell Signalling Technology (2729)

Supplementary Table IV

Customised primers used in this study from Integrity DNA Technology (IDT).

Customised primer		Sequences
Mm_Sm-mhc (Myh11)	Forward	5' - GCA GTG AGC TCT CAG TCA TC - 3’
	Reverse	5' - CAA TGC CTC CTC TGA CAA GT - 3'
Mm_Cnn1	Forward	5’ - GCT TGT CTG CTG AAG TAA AGA AC - 3'
	Reverse	5’ - TCC ATG AAG TTG TTC CCG ATG - 3'
Mm_Gli1	Forward	5' - TTG GAT TGA ACA TGG CGT CT - 3'
	Reverse	5' - CCT TTC TTG AGG TTG GGA TGA - 3'
Mm_Hprt	Forward	5’ - GGC TAT AAG TTC TTT GCT GAC CTG C - 3'

	Reverse	5’ - GCT TGC AAC CTT AAC CAT TTT GGG - 3'
Mm_Gaphd	Forward	5’ - GCC TCC AAG GAG TAA GAA AC - 3'
	Reverse	5’ - GCC TCC AAG GAG TAA GAA AC - 3'
Mm_ Sm-mhc	Forward	5’ - CCC TCC CTT TGC TAA ACA CA - 3'
(Myh11) for ChIP PCR	Reverse	5’ - CCA GAT CCT GGG TCC TTA CA - 3'

Primers used in this study from QIAGEN.

Primer	Product Name	Product Code
mHprt-1	Mm_Hprt_1_SG QuantiTect Primer Assay	QT00166768
mS100β	Mm_S100β_1_SG QuantiTect Primer Assay	QT00151536
mSox10	Mm_Sox10_1_SG QuantiTect Primer Assay	QT00295204
mNestin	Mm_Nes_1_SG QuantiTect Primer Assay	QT00316799
mGapdh	Mm_Gapdh_3_SG QuantiTect Primer Assay	QT01658692
mPax6	Mm_Pax6_1_SG QuantiTect Primer Assay	QT01052786
mPax1	Mm_Pax1_1_SG QuantiTect Primer Assay	QT01052779
mKdr	Mm_Kdr_1_SG QuantiTect Primer Assay	QT00097020
mTbx6	Mm_Tbx6_1_SG QuantiTect Primer Assay	QT00098861
hCNN1	Hs_CNN1_1_SG_QuantiTech Primer	QT00067718
hHPRT1	Hs_HPRT1_1_SG QuantiTect Primer	QT00059066
hMYH11	Hs_MYH11_1_SG QuantiTech Primer	QT00069391
hS100β	Hs_S100B_1_SG QuantiTect Primer Assay	QT00059164
rCnn1	Rn_Cnn1_1_SG QuantiTect Primer	QT01081115
rGli1	Rn_Gli1_1_SG QuantiTect Primer	QT01290324
rGapdh	Rn_Gapd_1_SG QuantiTect Primer	QT00199633
rHprt-1	Rn_Hprt1_1_SG QuantiTect Primer	QT00199640

Supplementary Table V

Antibodies and their corresponding dilutions used in Flow Cytometry.

Antibody/Product Name	Supplier/Product Number	Dilutions
Rat anti-mouse Sca1 (Ly-6A/E) [E13-161.7]	STEMCELL Technology (60032)	1/100
Rat anti-mouse IgG2a, kappa Isotype [RTK2758]	STEMCELL Technology (60076)	1/100
Rabbit Anti-mouse/rat S100β [EP1576Y]	Abcam (ab52642)	1/100
Normal Rabbit IgG (F graded)	Cell Signalling Technology (2729)	1/100
Alexa Fluor 647 Goat anti-mouse (H+L)	Life Technologies (A- A-21235)	1/100
