# Supplementary material for: The calcium binding protein S100β marks hedgehog-responsive resident vascular stem cells within vascular lesions
Source: NPJ Regen Med. 2021 Mar 1;6:10. doi: 10.1038/s41536-021-00120-8 (PMC7921434; doi:10.1038/s41536-021-00120-8)
Supplement: Supplementary file 2 — Supplentary Tables [file 41536_2021_120_MOESM2_ESM.pdf]

Supplementary Figures

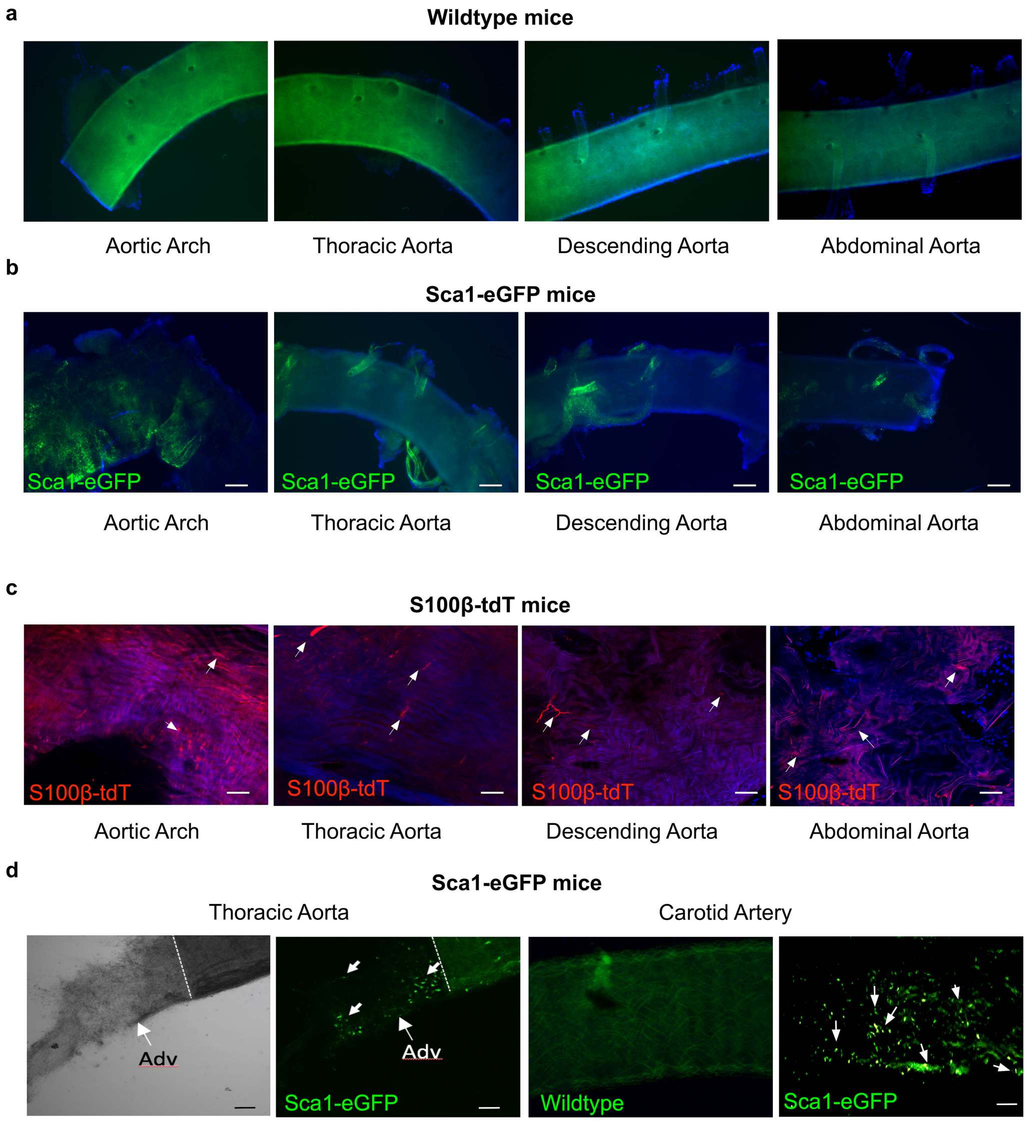

Supplementary Fig 1 (related to Fig 1). Sca1 and S100β cells are present in perivascular regions of murine arteries.

Whole mount analysis of **a.** wildtype control**, b.** Sca1-eGFP expression and **c.** S100β-Cre-ER2-tdT expression in aortic arch, thoracic aorta, descending aorta and abdominal aorta. **d.** Sca1-eGFP expression in mouse aorta following removal of the aortic media (dashed line) and in carotid artery of Sca1-eGFP transgenic mice. Each image is representative of 5 images from 3 animals.

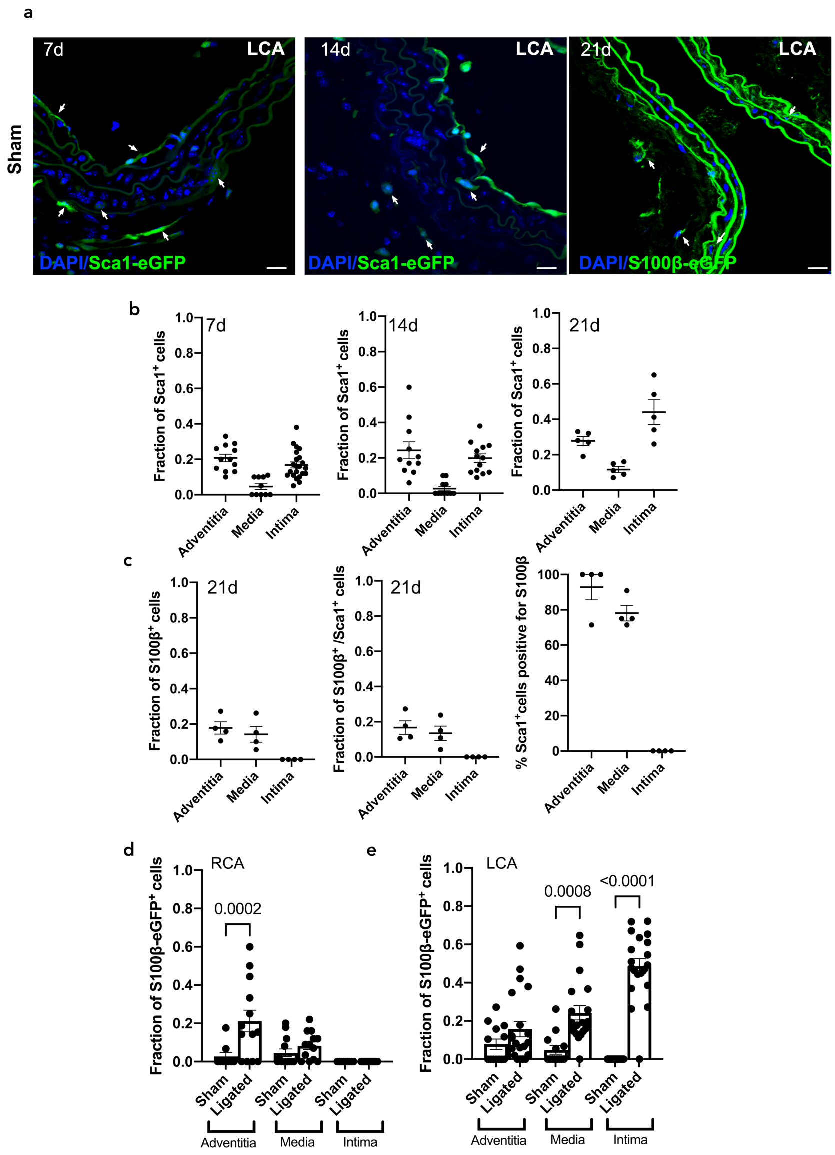

Supplementary Fig 2 (related to Fig 2). **Sca1 and S100β cells in adventitial, medial and luminal layers of normal murine arteries**.

a. Representative confocal images of Sca1^{+}-eGFP cells in the adventitial, medial and intimal layer of normal sham vessels after 7 days (right panel) and 14 days (middle panel) and S100β-eGFP^{+} cells after 21 days (right panel). **b.** Cumulative analysis of the fraction of Sca1^{+} cells in the adventitial, medial and intimal layer of sham vessels after 7, 14 and 21 days. Data are the mean ± SEM, n=5-15 sections from 3 animals/group. **c.** Cumulative analysis of the fraction of S100β^{+} cells, the fraction of S100β/Sca1^{+} cells and the percentage of Sca1 cells positive for S100β in the adventitial, medial and intimal layer of sham vessels after 21 days. Data are the mean ± SEM, n= 3 animals/group. Cumulative analysis of the fraction of S100β-eGFP^{+} cells in the adventitial, medial and intimal layer of **d.** RCA and **e.** LCA vessels, respectively, after 21 days in S100β-eGFP mice. Data are the mean ± SEM, n= 15-20 sections from 3-6 animals/group.

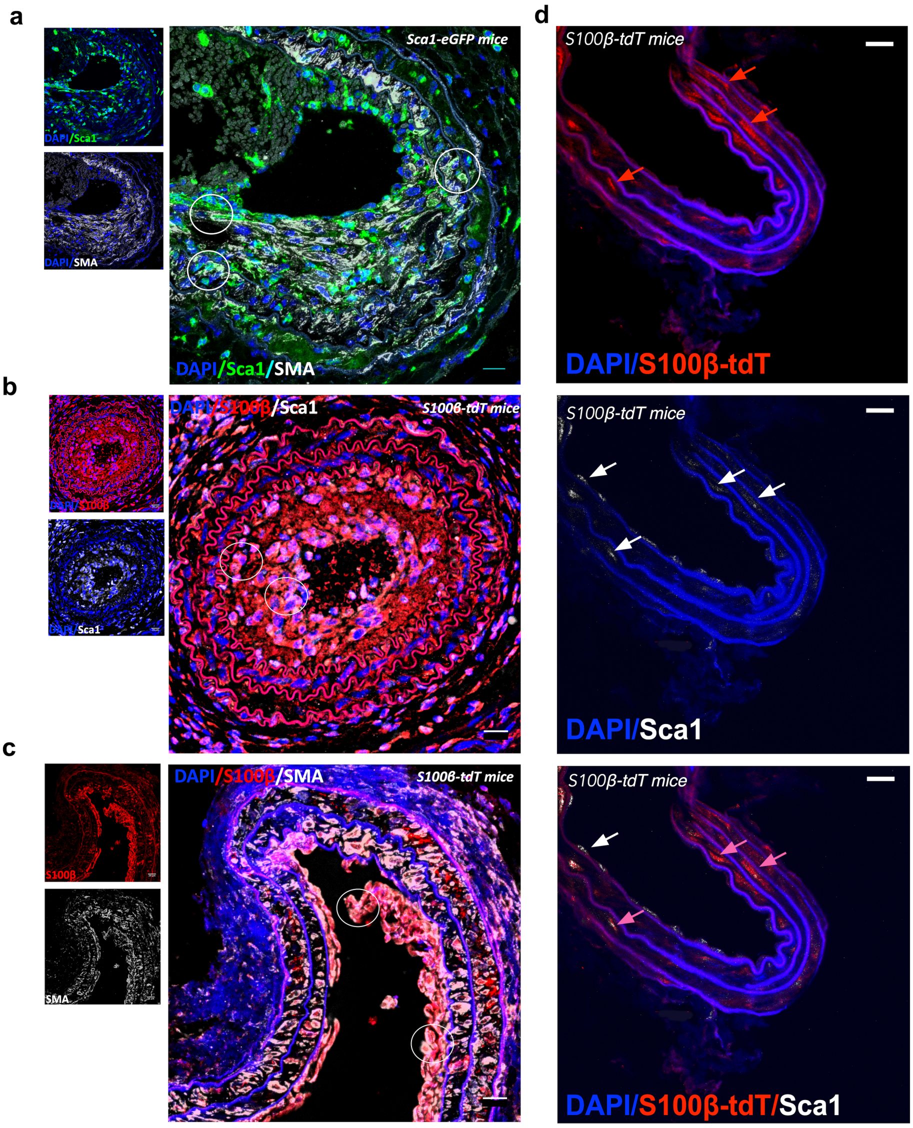

Supplementary Fig 3 (related to Fig 3). Co-localisation of Sca1, S100β and SMA cells in adventitial, medial and luminal layers of injured murine arteries.

a. Representative images of Sca1 (green, left top panel) and SMA (white, left bottom panel) and double stained Sca1/SMA cells (right panel) in ligated LCA of Sca1-eGFP mice after 14 days. **b.** Representative image of S100β-tdT (red, left top panel) and Sca1 (white, left bottom panel) and double stained S100β/Sca1 cells (right panel) of ligated LCA in S100β-CreERT2-tdT mice after 21 days. **c.** Representative image of S100β-tdT (red, left top panel) and SMA (left bottom panel) and double stained S100β/SMA cells (right panel) of ligated LCA in S100β-CreERT2-tdT mice after 21 days. Circles are representative of co-localisation. **d.** Representative images of S100β-tdT (red, left panel), Sca1-Far Red (white, middle panel) and double stained S100β/Sca1 (right panel) in the contralateral RCA after 21 days. Data are representative of 6 animals/group.

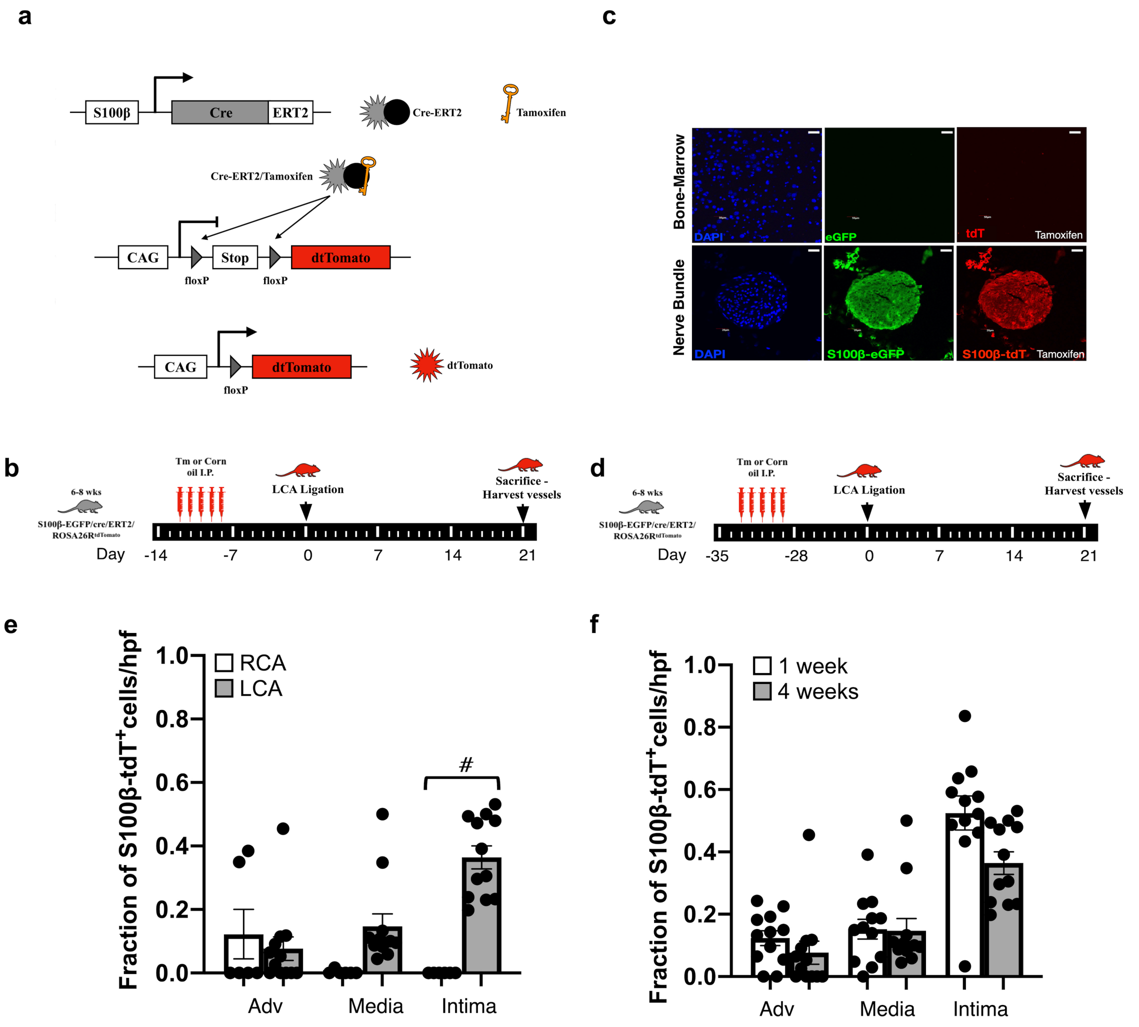

Supplementary Fig 4 (related to Fig 3). Lineage tracing analysis of S100β stem cells

a. Schematic diagram showing generation of S100β-CreERT2-Rosa26-tdTomato. **b**. Tm protocol of 1 week washout prior to ligation. **c.** Representative confocal fluorescence images of S100β-tdT expression in bone marrow smears and herrings nerve bundle in S100β-CreERT2-Rosa26-tdTomato **d.** Tm protocol of 4 week washout prior to ligation. **e.** The fraction of S100β-tdT cells within the adventitial, medial, intimal layers of LCA compared to RCA after 4 week washout. **f.** Comparison of the fraction of S100β-tdT cells within the adventitial, medial, intimal layers of LCA between 1 week and 4 week Tm washout protocols. Data are the mean ± SEM of 3-5 representative images from 3 animals, #p<0.05 vs RCA controls.

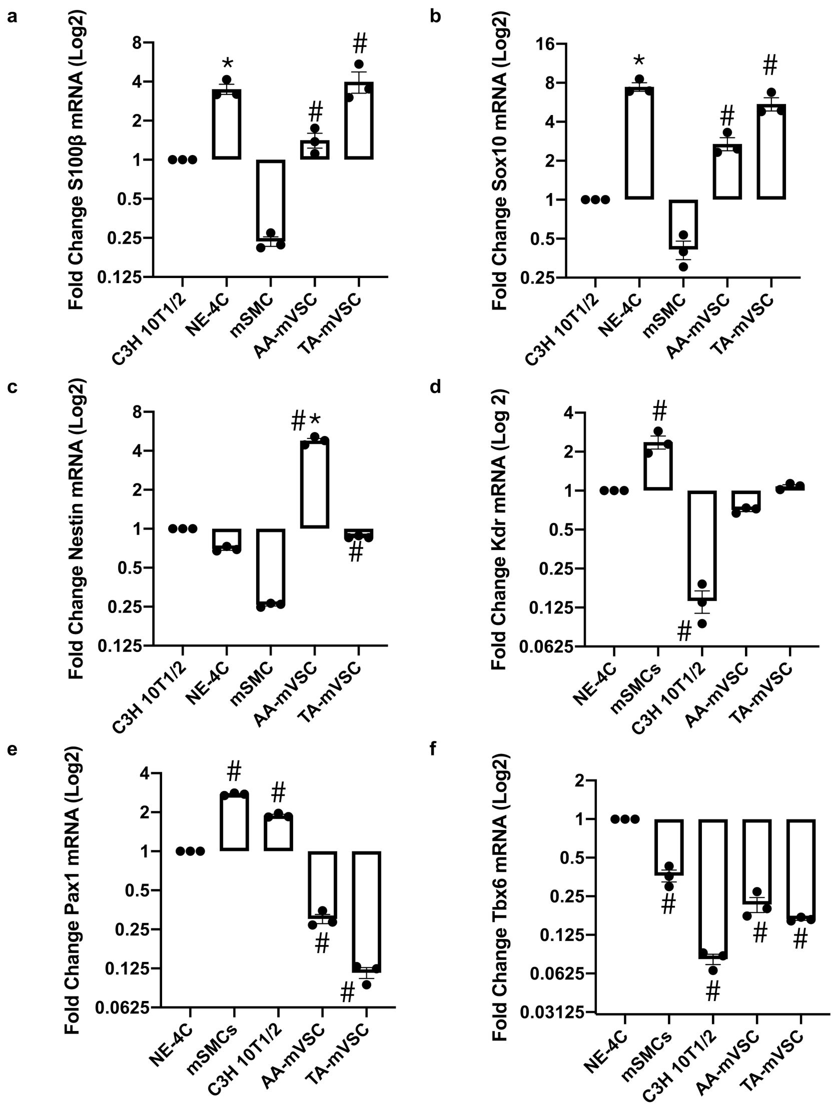

Supplementary Fig 5 (related to Fig 5). Neuroectoderm and paraxial mesoderm marker expression in murine vSCs *in vitro*

a-c. Relative levels of neuroectoderm markers (S100β, Sox10 and Nestin) and **d-f.** paraxial mesoderm markers (Kdr, Pax1 and Tbx6) in vSCs isolated from AA and TA regions of the mouse aorta. Data are expressed as the Log2 fold change in mRNA levels relative to C3H 10T1/2 cells or neural stem cells (NE-4C) in culture and are the mean ± SEM of 3 independent cultures, *p≤ 0.05 vs C3H 10T1/2 cells (a-c), #p≤ 0.05 vs NE-4C (d-f).

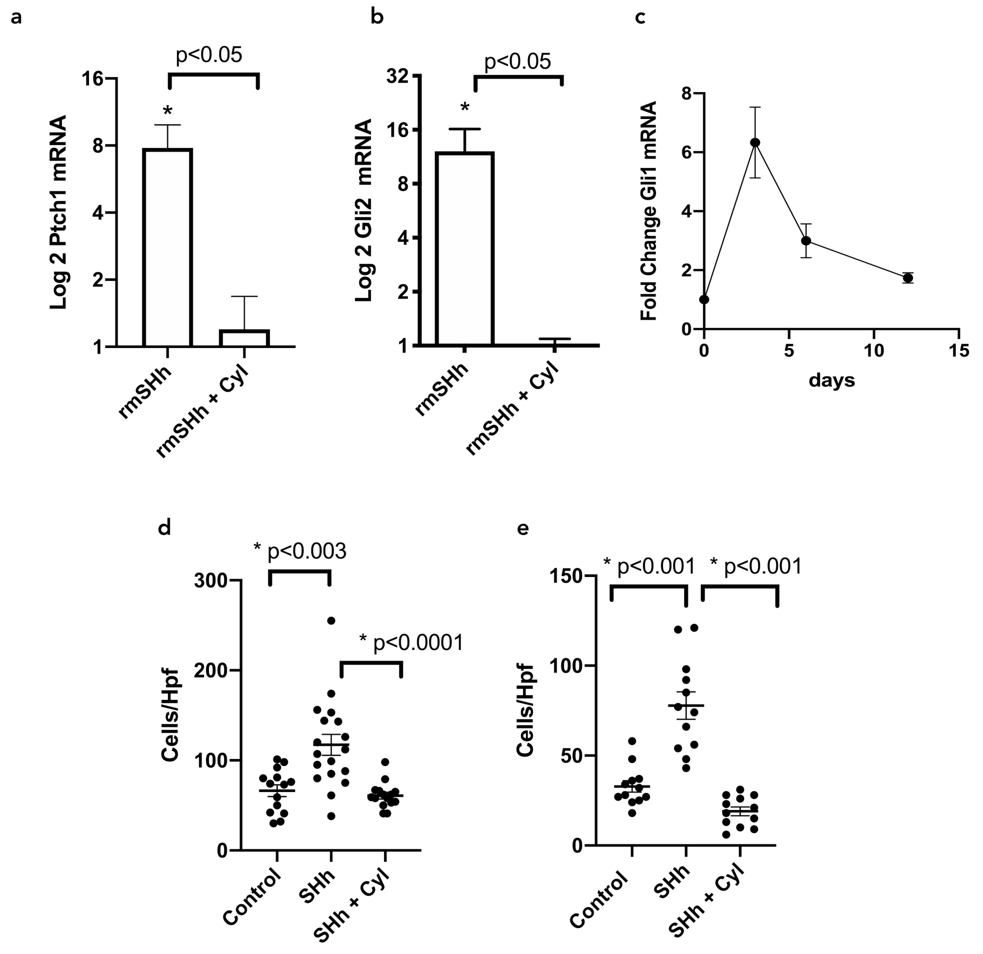

Supplementary Fig 6 (related to Figs 4 and 6). Hedgehog target gene expression and growth response to recombinant SHh.

Relative levels of **a.** Ptch1 and **b.** Gli2 in vSCs in the presence of rSHh (0.5 µg/ml) with or without the smoothened inhibitor, cyclopamine (10µM). Data are expressed as the Log2 fold change in mRNA levels relative to vSCs alone (control) and are the mean ± SEM of three representative wells, *p≤0.05 versus control. **c.** Temporal fold increase in Gli1 mRNA levels following carotid ligation. Data are the mean ± SEM from 3 vessels. The effect of recombinant SHh on the growth of **d.** S100β$^+$ vSC and **e.** S100β$^+$ NEPs in culture after 7 and 12 days, respectively. Data are the mean ± SEM of 8-12 wells/group.
