# Supplementary material for: The calcium binding protein S100β marks hedgehog-responsive resident vascular stem cells within vascular lesions
Source: NPJ Regen Med. 2021 Mar 1;6:10. doi: 10.1038/s41536-021-00120-8 (PMC7921434; doi:10.1038/s41536-021-00120-8)
Supplement: Supplementary file 3 — Reporting Summary Checklist [file 41536_2021_120_MOESM3_ESM.pdf]

nature research

Corresponding author(s): Paul A Cahill

Last updated by author(s): 08-01-2021

Reporting Summary

Nature Research wishes to improve the reproducibility of the work that we publish. This form provides structure for consistency and transparency in reporting. For further information on Nature Research policies, see our Editorial Policies and the Editorial Policy Checklist.

Statistics

For all statistical analyses, confirm that the following items are present in the figure legend, table legend, main text, or Methods section.

n/a	Confirmed	
☐	☒	The exact sample size (*n*) for each experimental group/condition, given as a discrete number and unit of measurement
☐	☒	A statement on whether measurements were taken from distinct samples or whether the same sample was measured repeatedly
☐	☒	The statistical test(s) used AND whether they are one- or two-sided<br>*Only common tests should be described solely by name; describe more complex techniques in the Methods section.*
☒	☐	A description of all covariates tested
☐	☒	A description of any assumptions or corrections, such as tests of normality and adjustment for multiple comparisons
☐	☒	A full description of the statistical parameters including central tendency (e.g. means) or other basic estimates (e.g. regression coefficient) AND variation (e.g. standard deviation) or associated estimates of uncertainty (e.g. confidence intervals)
☐	☒	For null hypothesis testing, the test statistic (e.g. *F*, *t*, *r*) with confidence intervals, effect sizes, degrees of freedom and *P* value noted<br>*Give P values as exact values whenever suitable.*
☒	☐	For Bayesian analysis, information on the choice of priors and Markov chain Monte Carlo settings
☒	☐	For hierarchical and complex designs, identification of the appropriate level for tests and full reporting of outcomes
☒	☐	Estimates of effect sizes (e.g. Cohen's *d*, Pearson's *r*), indicating how they were calculated

Our web collection on statistics for biologists contains articles on many of the points above.

Software and code

Policy information about availability of computer code

Data collection

Provide a description of all commercial, open source and custom code used to collect the data in this study, specifying the version used OR state that no software was used.

Data analysis

Provide a description of all commercial, open source and custom code used to analyse the data in this study, specifying the version used OR state that no software was used.

For manuscripts utilizing custom algorithms or software that are central to the research but not yet described in published literature, software must be made available to editors and reviewers. We strongly encourage code deposition in a community repository (e.g. GitHub). See the Nature Research guidelines for submitting code & software for further information.

Data

Policy information about availability of data

All manuscripts must include a data availability statement. This statement should provide the following information, where applicable:

- Accession codes, unique identifiers, or web links for publicly available datasets
- A list of figures that have associated raw data
- A description of any restrictions on data availability

All data generated during and/or analysed during the current study are available from the corresponding author on reasonable request.

Field-specific reporting

Please select the one below that is the best fit for your research. If you are not sure, read the appropriate sections before making your selection.

☒ Life sciences ☐ Behavioural & social sciences ☐ Ecological, evolutionary & environmental sciences

For a reference copy of the document with all sections, see nature.com/documents/nr-reporting-summary-flat.pdf

Life sciences study design

All studies must disclose on these points even when the disclosure is negative.

Sample size	The sample sizes were determined based on the degree of effect of the parameters measured, and based on the reasonable number of animals obtained in the study analyzed via multiple comparison tests. Some transgenetic lines required large breeding schemes only to get the number of animals presented in the manuscript.
Data exclusions	Some data were excluded from the manuscript for the purpose of using those data for other papers and/or grant submissions.
Replication	Replication was successful.
Randomization	All experimental groups were arranged in a random manner based on randomized breeding schemes and culling animals after genotyping.
Blinding	Blinding was applied to the data analysis for immunohistochemical and immunocytochemical analysis. Postgraduate students in the lab were asked to take measurements of samples without any indicators of origin, treatment or genotype.

Reporting for specific materials, systems and methods

We require information from authors about some types of materials, experimental systems and methods used in many studies. Here, indicate whether each material, system or method listed is relevant to your study. If you are not sure if a list item applies to your research, read the appropriate section before selecting a response.

Materials & experimental systems

n/a	Involved in the study	
☐	☒	Antibodies
☐	☒	Eukaryotic cell lines
☒	☐	Palaeontology and archaeology
☐	☒	Animals and other organisms
☒	☐	Human research participants
☒	☐	Clinical data
☒	☐	Dual use research of concern

Methods

n/a	Involved in the study	
☒	☐	ChIP-seq
☐	☒	Flow cytometry
☒	☐	MRI-based neuroimaging

Antibodies

Antibodies used

Antibodies and their corresponding dilutions used in immunohistochemistry.

Rat anti-mouse Sca1/Ly6A/E antibody [D7] (FITC) antibody Abcam (ab25031) 1/200
Rabbit anti-mouse/rat alpha smooth muscle cell alpha-actin antibody Abcam (ab5694) 1/200
Anti-Patched / PTCH1 antibody, Mouse monoclonal Abcam (Ab55629) 1/100
Anti-actin, α-Smooth Muscle antibody, Mouse monoclonal Sigma (A5228) 1/200
Anti-S100-β (CT) Antibody, clone EP1576Y, rabbit monoclonal Millipore (04-1054) 1/100
Anti-Gli2 antibody, rabbit polyclonal Novus Biologicals (NBP2-23602SS) 1/50
anti–alpha-smooth muscle actin (α-SMA) antibody (Abcam ab7817), 1/200
anti-eNOS antibody (Abcam ab76198) 1/200
anti-CD31 (Abcam ab24590) 1/100
Chicken anti-GFP antibody Abcam (ab13970) 1/500
Rabbit Anti-RFP/dT antibody Abcam (ab62341) 1/500
Goat anti-rabbit IgG secondary Alexa Fluor 647® conjugate Invitrogen (Cat # S32357) 1/1000

Antibodies and their corresponding dilutions used in immunocytochemistry

Mouse anti-mouse/rat nestin [Rat-401] Abcam (ab11306) 1/200
Rabbit anti-mouse Calponin [EP798Y] Abcam (ab46794) 1/200
Goat anti-mouse/rat/human smooth muscle Myosin heavy chain Santa Cruz (sc-79079) 1/200
Mouse anti-human/rat SOX10 R&D System (MAB2864) 1/100

Mouse anti-human SOX17 R&D System (MAB1924) 1/100
Rabbit Anti-S100β Merck Millipore (ABN59) 1/100
Rabbit Anti-mouse/rat S100β [EP1576Y] Abcam (ab52642) 1/100
Rabbit anti-mouse/rat Sca1 Millipore (AB4336) 1/100
Rabbit polyclonal anti-Oct4, Abcam (ab18976)1/100
Recombinant Anti-S100 beta antibody Abcam (ab52642) [EP1576Y]
Recombinant Anti-Calponin 1 antibody [EP798Y] (ab46794) 1/100
Alexa Fluor 488 Goat anti-mouse IgG Invitrogen (A-11001) 1/1000
Alexa Fluor 488 Goat anti-rabbit IgG Invitrogen (A-11008) 1/1000
Alexa Fluor 488 Donkey anti-goat IgG Invitrogen (A-11055) 1/1000

Antibodies used in Chromatin Immunoprecipitation (ChIP)
Rabbit anti-mouse Tri-Methyl-Histone H3 (Lys27) [C36B11] Cell Signalling Technology (9733S)
Rabbit anti-mouse Di-Methyl-Histone H3 (Lys4) [C64G9] Cell Signalling Technology (9725S)
Normal Rabbit IgG (ChIP graded) Cell Signalling Technology (2729)

Antibodies and their corresponding dilutions used in Flow Cytometry.
Rat anti-mouse Sca1 (Ly-6A/E) [E13-161.7] STEMCELL Technology (60032) 1/100
Rat anti-mouse IgG2a, kappa Isotype [RTK2758] STEMCELL Technology (60076) 1/100
Rabbit Anti-mouse/rat S100β [EP1576Y] Abcam (ab52642) 1/100
Normal Rabbit IgG (F graded) Cell Signalling Technology (2729) 1/100
Alexa Fluor 647 Goat anti-mouse (H+L) Life Technologies (A- A-21235) 1/100

Validation

For validation, we introduced background blocking controls as well as specificity controls that included secondary antibodies alone. These controls were compared to the primary antibodies and their staining patterns. Cell morphological features were also used to validate the staining as well. According to the manufacturers, each antibody was tested based on the above parameters using positive cell or tissue controls.

Eukaryotic cell lines

Policy information about cell lines

Cell line source(s)

Murine aortic SMCs (MOVAS (ATCC® CRL-2797™), Murine neuroectodermal stem cells (mNE-4Cs, ATCC® CRL-2925™), C3H 10T1/2 cells (ATCC® CRL-226™), Murine embryonic stem cells (mESCs) ES-D3 [D3] (ATCC® CRL-1934™), (HiPSC) were obtained from HipSci (Cambridge, UK) HPSI1013i-hiaf_1,

Authentication

Describe the authentication procedures for each cell line used OR declare that none of the cell lines used were authenticated.

Mycoplasma contamination

Confirm that all cell lines tested negative for mycoplasma contamination OR describe the results of the testing for mycoplasma contamination OR declare that the cell lines were not tested for mycoplasma contamination.

Commonly misidentified lines
(See ICLAC register)

Name any commonly misidentified cell lines used in the study and provide a rationale for their use.

Animals and other organisms

Policy information about studies involving animals; ARRIVE guidelines recommended for reporting animal research

Laboratory animals

Sca1-eGFP transgenic mice were obtained from JAX labs; Stock #012643, strain name B6.Cg.Tg(Ly6a-EGFP)G5Dzk/j. These transgenic mice have an enhanced green fluorescent protein (eGFP) under the control of murine lymphocyte antigen 6 complex, locus A (Ly6a) promoter. Hemizygous Ly6a-GFP mice are viable, fertile, normal in size and do not display any gross physical or behavioural abnormalities (Ma et al., 2002b).

S100β-EGFP/Cre/ERT2 transgenic mice (JAX Labs, stock #014160, strain name B6;DBA-Tg(S100β-EGFP/cre/ERT2)22Amc/j) express the eGFPCreERT2 (Enhanced Green Fluorescent Protein and tamoxifen inducible cre recombinase/ESR1) fusion gene under the direction of the mouse S100β promoter.

Ai9 mice (Jax Labs, stock #007909, strain name B6.Cg-Gt(ROSA)26Sortm9(CAG-tdTomato)Hze /J) express robust tdTomato fluorescence following Cre-mediated LoxP recombination.

For lineage tracing experiments S100β-eGFP/Cre/ERT2–dTomato double transgenic mice of both genders were generated by crossing S100β-eGFP/Cre-ERT2 mice with Ai9 reporter mice. The tdTomato transgene expression pattern corresponds to genomic marked S100β cells, and the eGFP transgene expression pattern corresponds to constitutive expression of S100β. Mice were genotyped using genomic DNA prepared from tail samples. All male and female mice were included in the study and were 8-10 weeks old

Wild animals

Provide details on animals observed in or captured in the field; report species, sex and age where possible. Describe how animals were caught and transported and what happened to captive animals after the study (if killed, explain why and describe method; if released,

	say where and when) OR state that the study did not involve wild animals.
Field-collected samples	*For laboratory work with field-collected samples, describe all relevant parameters such as housing, maintenance, temperature, photoperiod and end-of-experiment protocol OR state that the study did not involve samples collected from the field.*
Ethics oversight	Part of the animal studies were approved by The Jackson Laboratory Animal Care and Use Committee (Permit Number: 07007) and were in accordance with the "Guide for the Care and Use of Experimental Animals" established by the National Institutes of Health (1996, revised 2011). Part of the animal studies were also approved by the the University of Rochester Animal Care Committee in accordance with the guidelines of the National Institutes of Health for the Care and Use of Laboratory Animals.

Note that full information on the approval of the study protocol must also be provided in the manuscript.

Flow Cytometry

Plots

Confirm that:

☒ The axis labels state the marker and fluorochrome used (e.g. CD4-FITC).

☒ The axis scales are clearly visible. Include numbers along axes only for bottom left plot of group (a 'group' is an analysis of identical markers).

☒ All plots are contour plots with outliers or pseudocolor plots.

☒ A numerical value for number of cells or percentage (with statistics) is provided.

Methodology

Sample preparation	Flow cytometry was used to screen murine vascular stem cells isolated form aortic arch and thoracic aorta following expansion of these cells in culture. Fixation and permeabilisation was performed according to BD Bioscience Cytofix/ Cytoperm protocols. Briefly, 500,000 cells were used per sample. After the cells were pelleted they were fixed using BD cytoperm solution for 20 minutes at 4°C and permeabilised (if appropriate for the antigen). Following two brief wash steps the appropriate primary antibody was added. The cells were incubated at 4°C for 30 minutes. The cells were washed again and incubated with appropriate secondary antibody (Alexa Fluor 647) for 30 minutes at 4°C.
Instrument	BD FACSAria II and Amins Cell Stream
Software	FACS data analyzed using FlowJo™ software (Tree Star, Ashland, Ore) and De Novo software FCS Express 4 Flow Cytometry (Pasadena, CA) and Amnis CellStream® Acquisition and Analysis Software.
Cell population abundance	Appropriate controls were included including running S100B (NE4C neuronal cells) and Sca1 (mesodermal C3H 10T 1/2 cells) positive control cell lines and using isotype control IgG's for each antibody.
Gating strategy	Gating was based on using anti-S100B and anti-Sca1 antibodies added to cells using rat anti-mouse Sca1 (Ly-6A/E) [E13-161.7] STEMCELL Technology (60032) 1/100, rat anti-mouse IgG2a, kappa Isotype [RTK2758] STEMCELL Technology (60076) 1/100 rabbit Anti-mouse/rat S100β [EP1576Y] Abcam (ab52642) 1/100, normal Rabbit IgG (F graded) Cell Signalling Technology (2729) 1/100 and secondary antibody Alexa Fluor 647 Goat anti-mouse (H+L) Life Technologies (A- A-21235) 1/100 in staining buffer.<br><br>Cell populations were identified based on their size and their granularity which was determined by forward scatter (FSC) versus side scatter (SSC) respectively. As each cell type might have different size and granularity properties, different gating was applied to identify the cell population and to ensure the gating was still valid for each sample and was adjusted if necessary.<br><br>In addition to FSC vs SSC gating, cells were gated based on FSC-H and FSC-A light scatter properties and a gate was drawn to include the single cell population and to eliminate doublets. The single cells were selected for further analysis.<br><br>The singlets were then plotted in a single dimension to produce univariate histograms and bivariate contour plots. A positive and negative region was selected on a histogram for expression markers. The negative region covered the unstained cells and those stained with an Isotype control with a secondary antibody conjugated to AF647. The positive region covered the single cell population which expressed S100[illegible]1 or Sca1.<br><br>In bivariate contour plots, FSC was displayed on the y-axis and the S100[illegible]1 or Sca1 was plotted on the x-axis, and the cell counts or events are displayed as a density map.

☐ Tick this box to confirm that a figure exemplifying the gating strategy is provided in the Supplementary Information.
